# Early prediction of diagnostic-related groups and estimation of hospital cost by processing clinical notes

**DOI:** 10.1038/s41746-021-00474-9

**Published:** 2021-07-01

**Authors:** Jinghui Liu, Daniel Capurro, Anthony Nguyen, Karin Verspoor

**Affiliations:** 1 School of Computing and Information Systems, The University of Melbourne, Melbourne, VIC Australia; 2 Australian e-Health Research Centre, CSIRO, Brisbane, QLD Australia; 3 Centre for Digital Transformation of Health, Melbourne Medical School, The University of Melbourne, Melbourne, VIC Australia; 4 School of Computing Technologies, RMIT University, Melbourne, VIC Australia

**Keywords:** Health care economics, Computer science

## Abstract

As healthcare providers receive fixed amounts of reimbursement for given services under DRG (Diagnosis-Related Groups) payment, DRG codes are valuable for cost monitoring and resource allocation. However, coding is typically performed retrospectively post-discharge. We seek to predict DRGs and DRG-based case mix index (CMI) at early inpatient admission using routine clinical text to estimate hospital cost in an acute setting. We examined a deep learning-based natural language processing (NLP) model to automatically predict per-episode DRGs and corresponding cost-reflecting weights on two cohorts (paid under Medicare Severity (MS) DRG or All Patient Refined (APR) DRG), without human coding efforts. It achieved macro-averaged area under the receiver operating characteristic curve (AUC) scores of 0·871 (SD 0·011) on MS-DRG and 0·884 (0·003) on APR-DRG in fivefold cross-validation experiments on the first day of ICU admission. When extended to simulated patient populations to estimate average cost-reflecting weights, the model increased its accuracy over time and obtained absolute CMI error of 2·40 (1·07%) and 12·79% (2·31%), respectively on the first day. As the model could adapt to variations in admission time, cohort size, and requires no extra manual coding efforts, it shows potential to help estimating costs for active patients to support better operational decision-making in hospitals.

## Introduction

The payment system based on diagnosis-related groups, or DRGs, was designed to manage healthcare costs and maintain sustainable operations for inpatients and it has become a significant component of healthcare payments in many countries to promote risk-sharing between healthcare providers and payers^1,2^. The DRG system classifies inpatients with similar clinical and treatment characteristics into groups, where patients in the same group are expected to use similar amounts of resources, thus incentivizing providers to enable effective cost management. Hospital operation managers may review quarterly or even monthly DRG-based statistics to assess its patient mix^3,4^ and financial efficiency^5,6^ under DRG reimbursement. Meanwhile, such review is limited to retrospective assessment since DRGs are typically obtained after patient discharge, making it impossible to act upon this information to make adjustments for active patients. Furthermore, calculating DRGs is a time-consuming process requiring expert efforts to manually identify information from patient records, standardize it to ICD (International Classification of Diseases) format, and then obtain DRGs.

The rapid spread of electronic health records (EHRs) has created large amounts of patient data and provides an opportunity to estimate DRGs and related costs at early patient admission using machine learning. A prior study^7^ applied statistical machine learning for early DRG prediction and resource allocation at a 350-bed hospital in Germany, examined a range of feature selection and classification techniques in different prediction settings, and showed their DRG predictions were relevant for optimizing scarce hospital resources allocation and the improvement of contribution margin for the hospital. However, the study utilized derived patient-related data, including coded diagnosis and approximated DRG results, and evaluated the model with hospital-specific information on resources. In this study, we hypothesized that by processing routine clinical text with a deep learning-based model for active patients in the hospital, an automatic system can bypass the labor-intensive coding process that usually happens post-discharge and provide assistance to estimate hospital cost and case mix in a scalable way, thus supporting administrative decisions in real time. By leveraging a large set of patient data, deep neural network models have the potential to identify important diagnostic indicators from the raw data and encapsulate clinical patterns^8–10^. Since each DRG group corresponds to a defined weight representing the expected payment, the modeling results can be applied to estimate DRG-based inpatient cost at the hospital level. We developed a deep learning-based natural language processing (NLP) model on ICU patients using MIMIC-III^11^ for early classification of two DRG systems, namely Medicare severity-DRG (MS-DRG) and all patient refined-DRG (APR-DRG), which was subsequently applied to estimate cost for patient populations and assessed its potential to provide cost indicators, such as case mix index (CMI), for hospital administration.

## Results

### Study Cohort

Statistics on the datasets are presented in Table 1 for both MS-DRG and APR-DRG cohorts. For cost estimation on hospital populations, we kept only the first hospital visit of the patient in the test set to form the patient group. MS-DRG test cohort was reduced to 1648 hospital stays and APR-DRG test cohort to 2252. If a patient had both an MS-DRG and an APR-DRG, which sometimes may happen due to certain insurance policies, the patient was assigned to both cohorts. As mentioned previously, each unique DRG code in the APR-DRG cohort is an explicit combination of DRG group and patient severity that is assigned with a cost weight. The three most common major diagnostic categories (MDCs) in the two cohorts are diseases and disorders of the circulatory system (05), the nervous system (01), and the respiratory system (04).

**Table 1:** Statistics for the two datasets on DRG.

	Train set	Test set	Test cohort
MS-DRG			
Patient	14,836	1648	1648
Hospital stay	17,815	1977	1648
Unique DRG	595	369	349
DRG weight	3·045 (2·747)	3·049 (2·790)	3·147 (2·835)
Stay of MDC 05	4633 (26·0%)	522 (26·4%)	456 (27·7%)
Stay of MDC 01	2765 (15·5%)	320 (16·2%)	293 (17·8%)
Stay of MDC 04	2115 (11·9%)	219 (11·1%)	169 (10·3%)
APR-DRG			
Patient	20,266	2252	2252
Hospital stay	24,667	2747	2252
Unique DRG	893	517	480
DRG weight	3·265 (3·166)	3·291 (3·211)	3·300 (3·153)
Stay of MDC 05	6473 (26·2%)	733 (26·7%)	613 (27·2%)
Stay of MDC 01	3556 (14·4%)	424 (15·4%)	379 (16·8%)
Stay of MDC 04	2881 (11·7%)	319 (11·6%)	239 (10·6%)

Data is reported in count (%) or mean (SD). DRG is assigned on the basis of hospital stay, and a patient can have more than one stay in both datasets. Each unique DRG code in MS-DRG corresponds to the DRG group, whereas in APR-DRG the DRG code combines clinical group and patient severity. All DRG codes have a relative weight that is connected to reimbursement. Three most common MDCs: diseases of disorders of the circulatory system (05), nervous system (01), and respiratory system (04).

*DRG* diagnostic-related group, *SD* standard deviation, *MS-DRG* Medicare severity-DRG, *APR-DRG* all patient refined-DRG, *MDC* major diagnosis category.

### DRG prediction

We first present the evaluation results of the NLP model, adjusted convolutional attention for multi-label classification (CAML)^12^, for the two cohorts in Table 2, where mean (standard deviation [SD]) on the hold-out test set are reported based on five different models obtained in cross-validation experiments. For both MS- and APR-DRG, the NLP model achieved macro-averaged area under the receiver operating characteristic curve (AUC) over 0·86 and micro-averaged AUC over 0·95. For each MDC subset, the model was examined only on test cases with DRGs belonging to the MDC, such as diseases and disorders of the circulatory system, showing the capacity of the model to distinguish between major cases while alleviating the impact of numerous negative samples when calculating AUC scores. The model was able to achieve macro-AUCs (SD) of 0·836 (0·016), 0·850 (0·011), and 0·833 (0·013) on MS-DRG for diseases and disorders of the circulatory system, nervous system, and respiratory system, respectively, and similarly 0·881 (0·003), 0·892 (0·004), and 0·759 (0·012) for APR-DRG. The performance was also evaluated using F1 scores, where the model obtained 0·270 (0·006) and 0·244 (0·005) of micro-F1 on all cases in the two cohorts. Meanwhile, when looking at common DRG codes that account of 80% of total test stays, the results improved to 0·329 (0·008) and 0·306 (0·004), respectively and would further boost when examining more frequent DRGs.

**Table 2:** Main results on DRG prediction.

	DRG set	MACRO-AUC	MICRO-AUC	MACRO-F1	MICRO-F1	Number of DRG targets	Number (%) of hospital stays
MS-DRG							
CAML with clinical text	All DRGs	0·871 (0·011)	0·956 (0·002)	0·084 (0·008)	0·270 (0·006)	369	1977 (100·0)
	MDC 05	0·836 (0·016)	0·974 (0·002)	0·141 (0·010)	0·382 (0·011)	67	522 (26·4)
	MDC 01	0·850 (0·011)	0·974 (0·001)	0·121 (0·008)	0·343 (0·016)	52	320 (16·2)
	MDC 04	0·833 (0·013)	0·971 (0·003)	0·112 (0·018)	0·294 (0·024)	34	219 (11·1)
	Top 80% cases	0·923 (0·005)	0·983 (0·001)	0·192 (0·012)	0·329 (0·008)	131	0·923 (0·005)
	Top 50 DRGs	0·940 (0·002)	0·991 (0·000)	0·320 (0·014)	0·436 (0·009)	50	1083 (54·8)
	Top 30 DRGs	0·943 (0·002)	0·993 (0·001)	0·395 (0·013)	0·502 (0·006)	30	842 (42·6)
LSTM with clinical measurements	All DRGs	0·819 (0·009)	0·940 (0·004)	0·041 (0·008)	0·183 (0·011)	369	1977 (100·0)
APR-DRG							
CAML with clinical text	All DRGs	0·884 (0·003)	0·963 (0·001)	0·069 (0·008)	0·244 (0·005)	517	2747 (100·0)
	MDC 05	0·881 (0·007)	0·986 (0·000)	0·116 (0·004)	0·333 (0·006)	87	733 (26·7)
	MDC 01	0·892 (0·004)	0·984 (0·001)	0·123 (0·019)	0·321 (0·019)	63	424 (15·4)
	MDC 04	0·759 (0·012)	0·963 (0·002)	0·096 (0·032)	0·193 (0·016)	47	319 (11·6)
	Top 80% cases	0·936 (0·002)	0·985 (0·000)	0·180 (0·013)	0·306 (0·004)	180	2197 (80·0)
	Top 50 DRGs	0·952 (0·001)	0·993 (0·000)	0·336 (0·005)	0·434 (0·008)	50	1285 (46·8)
	Top 30 DRGs	0·942 (0·002)	0·995 (0·000)	0·375 (0·008)	0·484 (0·009)	30	976 (35·5)
LSTM with clinical measurements	All DRGs	0·838 (0·017)	0·946 (0·006)	0·037 (0·004)	0·160 (0·012)	517	2747 (100·0)

AUC and F1 scores on individual DRGs were macro-averaged or micro-averaged within each fold of the experiments, then the results were summarized in mean (standard deviation) over the performances of five models on the hold-out test set. DRG sets refer to stays in the test set used for evaluation that were assigned with either all DRGs (the original test set) or certain subgroups of DRGs (subsets of the test set). The DRG subgroups include most frequent DRGs and DRGs in the three most frequent MDCs, namely diseases and disorders of circulatory system (MDC 05), nervous system (MDC 01), and respiratory system (MDC 04). The number of DRG targets counts the unique DRGs in the DRG category, followed by the number (%) of hospital stays in the test set that were covered in the evaluation. For the model using clinical measurements, we only reported results using all stays as comparison.

*DRG* diagnostic related group, *MS-DRG* Medicare severity-DRG, *APR-DRG* all patient refined-DRG, *AUC* area under the receiver operating characteristic curve, *MDC* major diagnosis category.

The results of the reference model trained on structured clinical measurements are also reported in Table 2. Trained on time-series constructed on 104 clinical measurements using long short-term memory (LSTM)^13^, this model achieved macro-AUC of 0·819 (0·009) and 0·838 (0·017) for MS-DRG APR-DRG on all DRG codes. Although strong performances were observed with structured data, the NLP-based model consistently performed superior than the model using structured clinical data (*p* value < 0.01 under two-sided *t*-test), with strongest contrast on F1 scores. Detailed results of LSTM on DRG subgroups are listed in Supplementary Table 3.

### CMI prediction

**Fig. 1:**
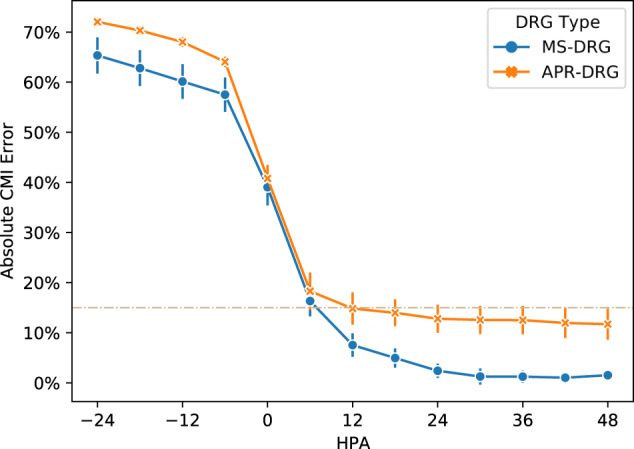
Absolute error on CMI across HPA. The plot shows the mean and standard deviation of absolute CMI errors at different HPA for MS-DRG test cohort (1648 patient stays) and APR-DRG test cohort (2252 patient stays), averaged over five models developed using different train folds. Predictions were made on the 6-h intervals from 24 h before ICU admission (−24 HPA) to 48 HPA. CMI case mix index, HPA hour post-admission.

The predicted CMI made by the NLP model on these two sets achieved errors of 2·40 (1·07%) and 12·79% (2·31%) on 24 h post-admission (HPA), or the first day of ICU, and tended to stabilize by 48 HPA (detailed numbers in Supplementary Table 2). Figure 1 shows how the CMI error changed with varying HPA, indicating the population progressed in the hospital stay. We included observations from a day before ICU admission (−24 HPA) to simulate the flow of patients through the ICU, which would include those that have already been admitted. The CMI prediction significantly improved around the ICU admission (−6 HPA and 6 HPA) and error reached lower than 5·0% for MS-DRG and 15·0% for APR-DRG after 24 HPA.

**Fig. 2:**
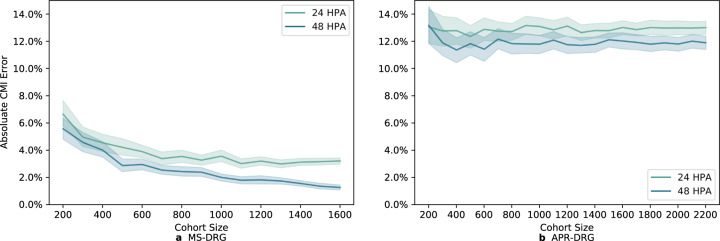
CMI for patient populations of different sizes. Performance to predict CMI (mean and 95% CI, based on 100 bootstrapped samples) on 24 and 48 h after ICU admission (24 and 48 HPA) for patient groups of different population sizes, ranging from 200 to 1600 for MS-DRG and 200 to 2200 for APR-DRG. Subfigure **a** shows results on the MS-DRG and **b** on the APR-DRG.

Besides admission time, we also examined how population size impacted the model on CMI prediction. For each population size, we randomly sampled 20 subgroups with replacement from the test cohort containing one visit per patient, and applied one of the five models obtained in the cross-validation experiments to predict CMI. This resulted in 100 subpopulations of the same size and the mean CMI and 95% confidence interval (CI) with bootstrapping were calculated at both 24 and 48 HPA on the two DRG types. Figure 2 demonstrates that at 24 HPA the model contained the CMI error under 8·0% for MS-DRG and 15·0% for APR-DRG with different cohort sizes, ranging from 200 to over 1500. Also, predictions made at 48 HPA tended to outperform those at 24 HPA.

**Fig. 3:**
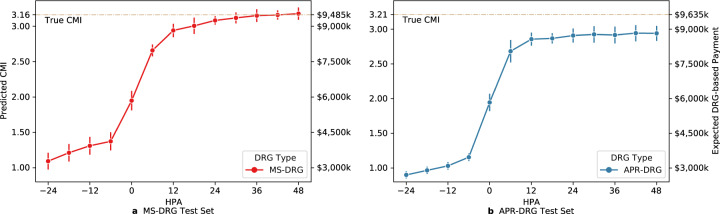
Predicted CMIs and related DRG-based payment amounts for a cohort of 500 stays. Predicted CMIs (instead of CMI errors) at different HPA on randomly sampled 500 hospital stays from each DRG cohort set, averaged over five models. Results on MS-DRG test cohort and APR-DRG test cohort are shown in subfigure **a** and **b**, respectively.

Finally, we mixed the two explorations on CMI above by randomly sampling 500 hospital stays from each DRG test set and presented predicted CMIs instead of absolute CMI errors. By assuming a base payment rate of $6000 and approximating DRG-based payment by the product of the rate and DRG weight, we could also estimate the DRG payment amount for a hospital. Figure 3 shows the model tended to underestimate population payments in the beginning and gradually became more reliable, with MS-DRG approaching true payment amount and APR-DRG stabilized at slightly lower level.

## Discussion

Healthcare expenditure has become a significant component of global spending^14^, and many health systems face challenges to improve efficiency and provide sustainable care^15,16^. Various payment incentives have been proposed and experimented with curbing the rising cost in the healthcare system to encourage healthcare providers to plan discreetly on resources^17,18^, promoting risk-sharing between payers and providers. Under prospective payment models, cost monitoring and management are vital for providers to stay financially competitive and are also essential for them to offer high-quality care. At the same time, precise cost information based on well-represented patient data may also assist payers in adjusting payment policies to further increase efficiency and support new payment initiatives^19,20^.

In this study, we focused on predicting episode-level DRG and estimating DRG-based expected payment at population level, which could help hospitals learn about their case mix and average cost at an early stage and make decisions accordingly. Existing studies^21,22^ examined health cost prediction from a payer perspective, such as using claims data to predict cost in subsequent years. Also, studies have shown the promise of machine learning in predicting events like unexpected 30-day readmission^8,23^ and identifying high-need high-cost patients^24^ that could preempt avoidable health expenses. Meanwhile, these studies did not focus on active patients to provide support for provider decisions. A prior study^7^ investigated DRG prediction in early hospital settings to enable better resource allocation, and it showed the machine learning-enabled approach could increase the contribution margin for the hospital. In our study, we developed deep learning-based models to process only routine clinical notes. The end-to-end modeling and the use of only routine patient data enables scalable and timely estimation at a population level to provide cost indicators, especially given that the standard calculation of DRGs involves intensive human effort and lengthy turn-around via manually coding ICDs. This is a major difference of our study on early DRG prediction as compared to the previous work including ICD codes in learning and prediction; ICD codes may not be readily available for all hospitals at early admission—at least not automatically. We also proposed to estimate hospital cost by predicting DRG-based payment at the hospital level instead of adopting hospital-specific evaluation.

Our study showed the feasibility of applying an NLP-based model to estimate the hospital cost based on DRG payment. DRG prediction using clinical text consistently performed better than clinical measurements alone, demonstrating the value of mining clinical text to support active care management. Though the deep learning-based NLP model could extract indicators to infer a reasonable DRG group for a patient stay, especially for diseases of circulatory system (MDC 05) and frequent DRGs, accurately predicting each individual DRG is still a challenging task. Error analysis showed the model correctly predicted many true negatives for each DRG given the class imbalance, thus increasing the average AUC performance, whereas the F1 scores reflect the errors on false positive and false negative predictions requiring future improvement. Meanwhile, at a population level, we showed that the model could achieve promising results when its predictions were translated to payment-reflecting weights and to reflect patient case mix. Population CMI, which averages over a set of patient DRG weights, is adopted by hospitals and payers as a cost indicator to review clinical complexity and efficiency. Our results showed the machine learning-based prediction could contain the CMI error under 8·0% for MS-DRG and 15·0% for APR-DRG on the first day of ICU admission. When calculating CMI, mistakes on individual cases offset each other to drive down the overall error, and we showed such performances were robust when the patient population size changed. Finally, performances in MS-DRG were in general better than APR-DRG, which could be due to APR-DRG having more fine-grained severity stratification and hence more prediction targets (1136 vs. 738).

Practically, early prediction of hospital CMI can provide valuable information on expected resource use, which can lead to better decision making in administration for improved care and managed cost^25^. Assuming a base payment rate of $6000 for the hospital, the CMI error rate of ~5·0% may lead to ~$472,500 difference in final reimbursement for a simulated cohort of 500 patients with a CMI of 3·15, but it allows the hospital to learn about its CMI at an early stage using only routine data and make decisions accordingly, implementing resource allocation and revenue capture. We observed better CMI performances for the MS-DRG cohort while the errors on APR-DRG were higher. Nevertheless, the model still achieved a mean below 15·0% and was more robust against changes in cohort size. The current examination of MS-DRG and APR-DRG showed the flexibility of the modeling approach on different DRG systems, which may be extended to other DRGs with similar structures of grouping and weight. Furthermore, it is possible to generalize the cost modeling strategy to scenarios other than DRG, where the model skips the DRG classification step and learns to predict patient cost in real numbers directly. We presented the approach in Supplementary Method 3 to apply the model to predict DRG payment weights in regression, where the model achieved mean absolute error (MAE) under 1·51 for both DRG cohorts (see Supplementary Table 1).

We should note that the current experimental results on CMI predictions show promise to provide management support at a hospital/population level rather than an individual/patient level. By forecasting the overall CMI of an in-hospital population, the model prediction may help administrators foresee peaks and troughs in resource usage and better arrange resources such as staffing and operating theaters. The current model performance in predicting individual DRGs is still inadequate to provide decision support on individual cases, which requires future research and additional consideration of ethical concerns.

Since the focus of this work is to examine the feasibility of DRG prediction and DRG-based cost estimation based on clinical notes, we did not examine the NLP methodologies comprehensively and regard such an investigation as future work. Meanwhile, we found CAML to be a simple yet effective baseline even compared against large pretrained models like BERT^26^ and ClinicalBERT^27^ (the domain adapted version of BERT), which provided improved AUC but lower F1 scores compared to CAML in both cohorts. Details of our experiments with these models are available in Supplementary Method 4 and results in Supplementary Table 4.

To better understand the challenges of text-based early DRG prediction and provide guidance for future methodological improvements, we selected five discrete hospital stays in the MS-DRG cohort to compare the model prediction with the true DRGs and focused on understanding the modeling errors. Here we leveraged the attention mechanism to identify the most informative tokens (in this case, 5 g) considered by the model in making its prediction to support the analysis (the role of attention to provide interpretability is still under active research^28,29^), shown in Table 3. We can observe that the model could extract the most indicative text when making the correct predictions in Case 1, including “*coronary artery bypass graft*” and “*cabg*”. Meanwhile, the model could fail at differentiating multiple diagnoses when faced with complex patient conditions. For example, in Case 2, the model correctly extracted text on pneumonia, thus classifying the case as DRG 193, but failed to recognize its diagnostic relation to more complicated conditions like sepsis. Finally, we should note that the attention mechanism here aims to support the analysis and its role to provide interpretability of the model is still under active research.

**Table 3:** Case analyses on DRG prediction.

Case (stay ID)	True DRG (case count in the cohort)	Predicted DRG (case count in the cohort)	Top *n*-grams	Attention weight
Case 1 (134,183)	236: Coronary bypass W/O cardiac cath W/O MCC (634)	236: Coronary bypass W/O cardiac cath W/O MCC (634)	…disease\coronary artery bypass graft /sda… …s/p cabg x 3 s… …artery bypass graft /sda respiratory…	0.9523 0.0076 0.0044
Case 2 (112,077)	871: Septicemia or severe sepsis W/O MV 96+ hours W MCC (968)	193: Simple pneumonia & pleurisy W MCC (210)	…<> pneumonia 5:20 pm chest… …admitting diagnosis: pneumonia medical… : 76 m with hypoxia reason…	0.6463 0.0691 0.0440
Case 3 (100,852)	025: Craniotomy & endovascular intracranial procedures W MCC (376)	026: Craniotomy & endovascular intracranial procedures W CC (136)	…s/p right craniotomy for tumor… …right brain mass/sda medical condition… …right cerebellar metastasis, presurgical…	0.1072 0.0723 0.0596
Case 4 (190,645)	955: Craniotomy for multiple significant trauma (21)	023: Cranio W major dev impl/acute complex CNS PDX W MCC or chemo implant (103)	…man s/p craniotomy and evacuation… …right frontotemporal craniotomy with evacuation… …s/p pedestrian struck medical condition…	0.4381 0.1583 0.0252
Case 5 (160,077)	289: Acute & subacute endocarditis W CC (1)	378: G.I. Hemorrhage W CC (327)	…gastrointestinal bleed 11:22 pm chest… …gastrointestinal bleed medical condition:… …: gastrointestinal bleed medical condition…	0.7385 0.0870 0.0715

Here we present five cases from the MS-DRG cohort to explore the predictions of the model and compare them with the expected DRGs. The stay ID of each case corresponds to the hospital admission ID. In addition to DRG code and description, we also count the number of DRG cases in the whole cohort. For each case, we present the top three *n*-grams (in this case 5-g) with the highest attention weights assigned by the model when making its decision.

The presence of comorbidity or complication (CC) or major CC (MCC) also introduces challenges to the model in recognizing and prioritizing diagnoses. Case 3 shows an example where the model was confused between DRGs with MCC and with CC.

In addition, the machine learning model could also suffer from a lack of enough training data and make errors on rare DRGs, such as the model predicted the more frequent DRG 023 for Case 4, whose true DRG is in fact 955 (both DRGs are related to craniotomy). Case 5 is an extreme case where DRG 289 only appeared once in the whole cohort, and it was in the test set, creating a zero-shot learning scenario for the model as it had not seen any training sample of the DRG.

Several limitations exist in our study for the early prediction of DRG-based hospital cost. First, we only examined data from one medical center in the United States. Though MIMIC-III contains a large number of patients and the study design is at hospital level, the dataset is still limited in location and patient representativeness. Due to the data de-identification, we were not able to apply DRG weight mapping to the exact fiscal year, so we used the official DRG weights published for fiscal year 2013 given MIMIC-III collected data until 2012. Second, the focus on ICU patients also leaves room for further exploration, including input data and predictor selection. On the one hand, the acute conditions of ICU patients are more dynamic and less predictable, and strong performances on them may indicate the capacity of the approach to model other inpatients, which should be studied. On the other hand, intensive care for ICU patients usually generates more data than other inpatients, providing more signals for a learning model. Sufficient data is vital for proper modeling and hence further investigation is necessary to explore the possible trade-off between disease severity and data availability on new patient populations.

Thirdly, though achieving favorable results on population CMI, the modeling method of clinical notes remains to be improved to make more accurate DRG predictions at an individual level. Early DRG prediction involves several challenges, including extracting diagnostic evidence, identifying major or negligible comorbidities, handling rare or unseen test DRGs, and modeling dynamic patient trajectories. This will require further innovations with consideration of the task and the characteristics of clinical text. As shown in the case analyses, CC and MCC play significant roles in the DRG payment but are challenging to differentiate based on text. The different clinical functions reflected in the texts could be one reason for the difficulty; for example, a radiology report can describe a radiograph for diagnostic purposes or for examination purposes (i.e., if catheterization is performed appropriately). Future work could consider modeling different types of clinical texts with separate modules instead of modeling the concatenated notes. This could also alleviate the impact of input length on Transformer-based models like BERT, which we believe was the main constraint for BERT to outperform the simpler CAML. As we also found domain adaptation improved BERT performances (see ClinicalBERT scores in Supplementary Table 4), a domain-specific efficient BERT, like Longformer^30^, may achieve much better performance on the task. In addition, the ontological structure of DRG could provide information to handle rare DRGs in few-shot and zero-shot learning scenarios, which are shown to be applicable in automatic ICD coding^31^. Finally, the efficacy of the NLP approach remains to be investigated in other data sets, including those in languages other than English.

## Methods

### Study design

Under the DRG payment system, an inpatient hospital admission is expected to receive a single DRG code under a specific DRG payment system to process claims with a payer. Two primary systems are MS-DRG used by the Centers of Medicare and Medicaid Services (CMS), and APR-DRG adopted by many private payers. Each DRG system includes two main components: the grouping logic to define the clinical boundaries and clinical severity of a case, and the relative weight to indicate the resource usage and consequent treatment cost for the DRG group. During payment, the DRG groups and their corresponding weights are pre-defined, so a provider can expect reimbursement for an assigned DRG by multiplying the relative weight by a fixed dollar amount, referred to as the base payment rate, which is specific to the provider based on local factors like wages.

We perform modeling and evaluation according to the two components of the DRG system, namely the DRG grouping and cost estimation in the form of DRG weights. We first developed the NLP model as a multi-class classifier trained for DRG grouping based on early patient notes. Then we obtained corresponding DRG weight for cost estimation based on the predicted grouping.

### Dataset and preprocessing

For the current experiments, we used the third version of Medical Information Mart for Intensive Care (MIMIC-III) that contains de-identified data from ICU patients at a major medical center in Boston, US. The data collection process and methods are described in detail in the original study^11^. We examined two versions of DRG available in our dataset, namely MS-DRG and APR-DRG, creating two DRG cohorts for model development and evaluation on active ICU patients. We constructed the cohort for each DRG system by selecting hospital stays assigned with the relevant DRG, including patients over 18 years of age and stays that involve only one ICU visit (Supplementary Fig. 1 and Supplementary Note 1).

Compared to MS-DRG, APR-DRG has an extra nuance in the grouping as each DRG group is further stratified into four subgroups by severity and mortality. Meanwhile, each subgroup in APR-DRG is still associated with a pre-defined weight. We therefore represented the subgroups with unique DRG codes in the experiments. In forming the target DRG space for model design, we excluded codes related to neonate or post-care, resulting in 738 codes for MS-DRG and 1136 for APR-DRG. Notice these numbers were based on the official DRG rules and were larger than the unique DRGs observed in the cohorts (611 and 908, respectively).

Patient notes were collected from the beginning of hospital admission, and were recorded relative to the time of ICU admission, referred to as HPA. We included only clinical notes charted up to 48 HPA, or 48 h after ICU admission. Note that a clinical note item can be charted at a negative HPA like *−*6, i.e., the 6th hour *before* ICU admission, due to the possible gap between hospital admission and ICU admission. We considered only hospital visits with at least one clinical note in the 48 HPA window for each DRG cohort. The choice of 48 HPA was to fit the early prediction scenario while providing sufficient data for learning.

As input data, clinical texts include nursing notes, physician notes, radiology reports, and other notes charted within the timeframe; reports created at a later stage, such as discharge summaries, were excluded. Clinical notes were sorted by chart time and concatenated to form a single text string per patient admission. We adopted pretrained word embeddings trained on large biomedical and clinical corpora from previous work^32^ and followed their steps to remove de-identification placeholders from the concatenated text and tokenize the sequence. Tokens were lowercased and those appearing at least three times in the cohort dataset were associated with pretrained embeddings. Infrequent tokens and tokens not found in the pretrained embeddings were mapped to one specific, randomly initialized embedding vector. Given the large variance in note length, we used the mean text length up to 48 HPA of 2000 tokens as the sequence length for the current experiment; each input is either padded with zero-vector or truncated to this length.

### Model development

The clinical text was modeled using a Convolutional Neural Network (CNN)^33^. When modeling sequential data, one-dimensional CNN can be understood intuitively as extracting predictive *n*-gram features and encoding these features in a latent representation. CNN filters slide across the text and produce corresponding feature maps, which are then pooled into downstream features. In this study, we adopted an architecture shown to be effective for automatic ICD coding named CAML^12^ that pools CNN features using the attention mechanism, modifying it for the single-label DRG prediction task. The model encodes text into feature vectors processed by a fully-connected neural network as classifier; each of its hidden states corresponds to one possible DRG code. To obtain outputs, we take the code with the highest probability as the result for DRG grouping and concurrently obtain the payment weight corresponding to the predicted DRG code. The mathematical formulation of the model and training objective are presented in Supplementary Method 1.

To compare the use of clinical text with other readily accessible, routinely collected data, we developed another neural network-based model for early prediction of DRGs using structured clinical measurements, such as vital signs and lab measurements, following a curated benchmark pipeline^34^. This provides a vigorous feature set for MIMIC-III data, constructing patient time-series with 104 clinically aggregated variables for an alternative model (see Supplementary Method 2 for the complete variable list). Implementation details of both text and measurement-based models are provided in Supplementary Note 2.

### Cross-validation and statistical analysis

We first kept 10% of data as the hold-out test set for each DRG system and then performed fivefold cross-validation, training, and tuning the hyper-parameters of independent models on five different splits of the remaining 90% of data. The performance of these five models on the test set were aggregated to report the mean and SD under a specific metric. The datasets were split by patient instead of by admission for both test set and cross-validation, ensuring that a patient would not appear in both cross-validation and testing and therefore avoiding data leakage. When evaluating cost estimation on simulated populations, the test set was further trimmed by keeping only one hospital admission for each unique patient to form the test cohort.

To evaluate DRG prediction performance, we calculated the AUC for each DRG code and aggregated the results via macro- and micro-averaging on the test set labels. We also computed F1-score in both macro and micro versions. Besides reporting performances on all test cases, we created subsets focusing on specific DRGs, including the most frequent DRGs and DRGs of three common MDCs based on body system or etiology. The metrics were averaged over the results from the five models on the hold-out test set and reported in mean (SD).

To evaluate DRG-based cost estimation for patient populations, we adopted the notion of the CMI^3,25^, which is an averaged score of DRG weights given a patient group. We computed the absolute CMI error by simply dividing the difference between the predicted CMI and the true CMI by the true CMI of the target population. This CMI error was further reported under two variations to simulate hospital scenarios. The first variation involved progressively changing the HPA to explore when the NLP model receives sufficient data to make useful cost estimation. Secondly, we down-sampled the unique patients in the test set to form smaller patient populations, aiming to explore whether the model is robust against changes in population size.

### Reporting summary

Further information on research design is available in the Nature Research Reporting Summary linked to this article.

## Supplementary information

Supplementary Information

Reporting Summary

## Data Availability

Access to MIMIC-III can be requested at https://physionet.org/content/mimiciii/1.4/, which requires signed safe usage agreement and for research-only.

## Code availability

Scripts for preprocessing, training, and evaluation in this work were written in Python, using packages including pytorch and scikit-learn. They are available with accompanied documentation at https://github.com/JHLiu7/EarlyDRGPrediction.
