# Supplementary material for: Early prediction of diagnostic-related groups and estimation of hospital cost by processing clinical notes
Source: NPJ Digit Med. 2021 Jul 1;4:103. doi: 10.1038/s41746-021-00474-9 (PMC8249417; doi:10.1038/s41746-021-00474-9)
Supplement: Supplementary file 1 — Supplementary Information [file 41746_2021_474_MOESM1_ESM.pdf]

Supplementary Material

Supplementary Figure 1. Dataset construction

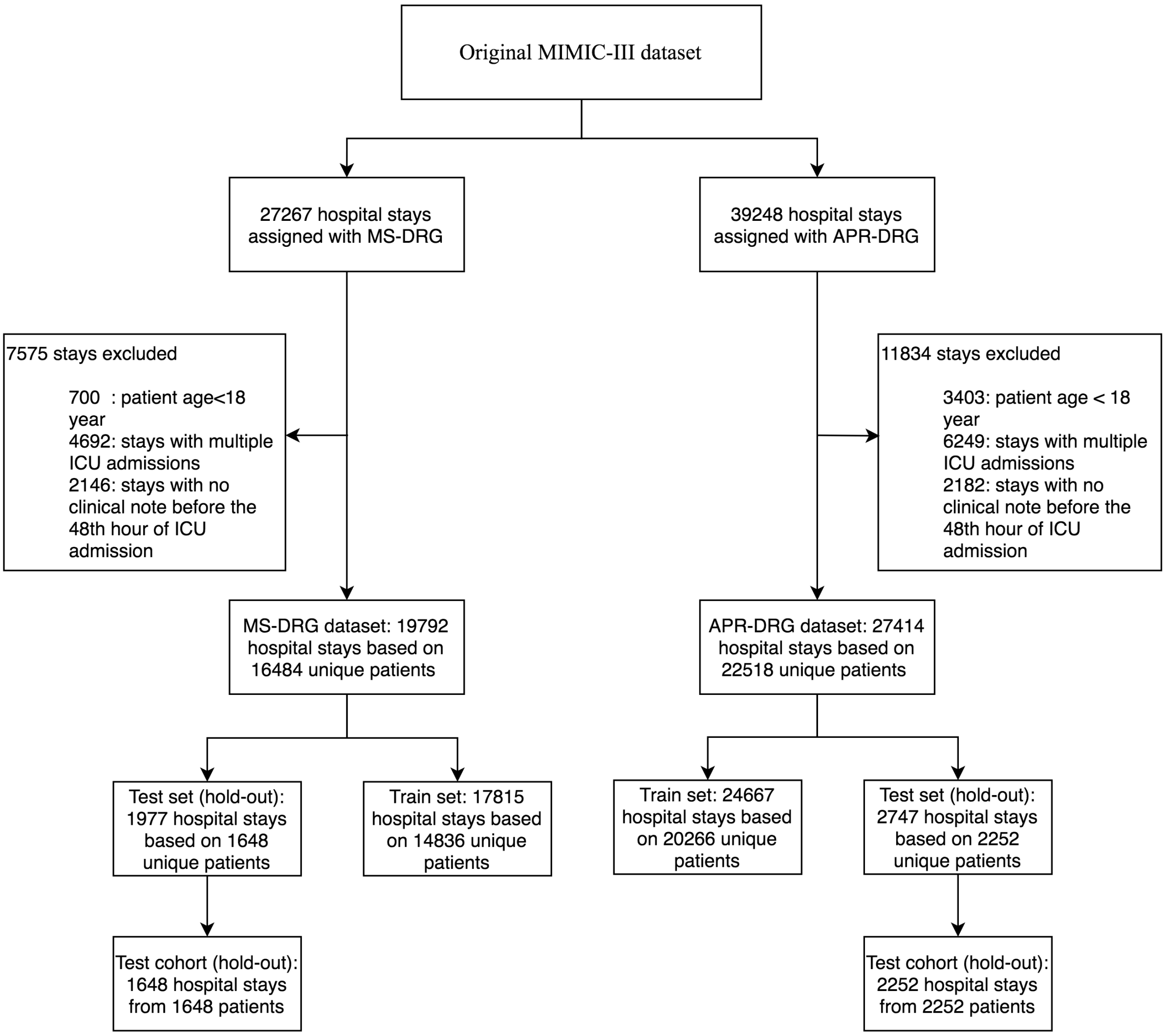

Processing steps and statistics for dataset construction.

Supplementary Note 1. Details on Dataset Construction

We constructed two datasets for the experiments using MIMIC-III, each focusing on a widely implemented DRG system, namely MS-DRG and APR-DRG. To select hospital admissions from the original dataset of MIMIC-III, we did a initial filtering on the stays assigned with DRG codes to avoid errors in the recording. For MS-DRG, we removed DRGs in MIMIC-III that do not have a DRG description of the group and dropped duplicated hospital stays. For APR-DRG, we additionally dropped the DRG codes if we found more than one APR-DRG group or more than one severity score assigned to the same APR-DRG group in each hospital stay. The initial filtering provided 27267 stays for MS-DRG and 39248 stays for APR-DRG, which were processed further by keeping only 1) adult patients (age $>$ 18 years), 2) hospital stays with only one ICU admission, 3) and stays with at least one clinical note charted before the 48th hour at ICU. The MS-DRG and APR-DRG datasets were then split into train set and hold-out test set for model derivation and validation, and the test set was trimmed to form a simulated test cohort by keeping only one hospital visit for each unique patient. The processes and statistics are presented in Supplementary Figure 1.

Cost weights for the DRG groups were obtained via official publications on governmental websites*, and the versions of fiscal year 2013 were used for both MS-DRG and APR-DRG. These documents lists all DRG groups and their corresponding relative weights based on which payers reimbursement hospitals. We denoted each DRG group as an independent target for MS-DRG and denoted a DRG concatenated with its severity score as a target for APR-DRG, because each DRG group in MS-DRG corresponds directly to a cost weight whereas in APR-DRG the weight maps to the combination of DRG group and severity. As an example for APR-DRG, DRG group 139 (Other Pneumonia) has four subgroups categorized by patient severity ranging from 1 to 4, each with a unique cost weight and thus can be treated as a unique DRG target. This enabled us to handle the extra component of patient severity and mortality in APR-DRG to focus on the final reimbursement rate. Then for each DRG dataset, we obtained the ground rules for weights that map a DRG target or DRG code $y^a \in Y$ (including its description) for patient p^a to a relative payment weight, which is denoted as $rw^a = drgRule(y^a)$.

Supplementary Method 1. Clinical text-based model

In this section we formalize the problem of DRG classification and weight prediction and describe the adopted modeling strategies. The deep learning architecture to model text was inspired by the work[1] for ICD-tagging. For our task on DRG prediction, we model the available data between the hospital admission and the T-th hour after ICU admission (which we refer as hours post admission (HPA)) of the patient p^a. We use such data as input to predict the DRG code obtained after patient discharge, which we denote as the target $y^a \in Y$ and can be mapped to a relative weight rw^a based on existing payment rules as described in the main article and the last section.

The clinical reports N_i assigned to a patient p^a before a given time threshold T_i are used in the modeling. The reports are tokenized and concatenated into a single text composed of words (tokens) $w_1, w_2, ..., w_n$. These words are then embedded with pre-trained word embeddings[2] to form the input representation of the clinical text, which we denote as $\mathbf{e}_{1:n} = [\mathbf{e}_1, \mathbf{e}_2, \dots, \mathbf{e}_n] \in R^{d^e \times n}$, with d^e being the embedding dimension. This represents the information about the early patient stay.

Subsequently, a number of one-dimensional convolutions are performed to adaptively extract phrases that are most informative for making predictions. Here the classic setting is to have convolutional filters with multiple feature maps as well as filters of different window sizes. Specifically, a filter f^j, composed with parameters $\mathbf{w}^j \in R^{u_i \times d^e}$ and $b^j \in R$, performs convolutions by rolling through the input sequence $\mathbf{e}_{1:n}$ with a fixed-sized text window, or kernel size, $u_i \in U$. This step produces a feature map $\mathbf{c}^j = [c_1^j, c_2^j, \dots, c_{n-u_i+1}^j]$, where

$$c_k^j = relu(\mathbf{w}_i \cdot \mathbf{e}_{k:k+u_i-1} + b) \tag{1}$$

and with F filters the whole convolution operation encode the text as $\mathbf{C}_T = [\mathbf{c}^1, \mathbf{c}^2, ..., \mathbf{c}^n] \in R^{|F| \cdot |U| \times n}$.

Latent text features can then be obtained either via max-pooling the feature map or by applying an attention mechanism. For attention-based feature pooling in CAML,[1] each label is assigned with a vector $\mathbf{v}_\ell$, which queries the features map matrix $\mathbf{C}_T$ to produce weight distribution over different e_i ($i \in n$),

$$\alpha = softmax(\mathbf{C}_T^\top \mathbf{v}) \tag{2}$$

where $softmax(\mathbf{x}) = \frac{exp(\mathbf{x})}{\sum_i exp(\mathbf{x}_i)}$.

Features for DRGs are then computed as $\mathbf{h}_{y_i} = \sum_{i=1}^n \alpha_{\ell,n} \mathbf{c}^i$ to form $\mathbf{H}_T = [\mathbf{h}_{y_1}, \mathbf{h}_{y_2}, \dots, \mathbf{h}_{y_Y}] \in R^{|F| \times |Y|}$. In CAML, this representation is then transformed with prediction weights to produce output $\mathbf{O}_T \in R^{|Y|}$ where $\mathbf{O}_{T,\ell} = \beta^\top \mathbf{v}_\ell + b_\ell$. Notice we do not apply sigmoid transformation here as in[1] since DRG prediction is a single-label classification, in contrast with the ICD prediction that CAML was designed originally to solve.

With that, the distribution of the probabilities for DRG codes is $\hat{y} = softmax(\mathbf{O}_T) \in R^{|Y|}$ and $|Y| = 745$ for our current experiment, one dimension per DRG code as stated in the article. The final DRG prediction for patient p^a with input data at the T-th hour of ICU is then $\hat{y}^a = argmax_i(\hat{y})$, and the relative weight prediction is $\hat{rw}^a = drgRule(\hat{y}^a)$.

To train the model for DRG classification, we minimize the cross entropy loss between the target DRG y and the predicted likelihood $\hat{y}$ as our training objective. If we parameterize the model by θ, the cross entropy loss is:

$$CrossEntropy(D, y, \theta) = -\sum_{i=1}^{|Y|} y_i log(\hat{y}_i) \tag{3}$$

and this is aggregated for all samples in a training batch to update θ with gradient method.

*MS-DRG: https://www.cms.gov/Medicare/Medicare-Fee-for-Service-Payment/AcuteInpatientPPS, APR-DRG: https://www.health.ny.gov/facilities/hospital/reimbursement/apr-drg/weights/

Supplementart Method 2. DRG Prediction using Structured data

Besides using clinical text, we also explored to use structured clinical measurements to make early DRG predictions in the ICU setting. A range of works in recent years have examined using rich measurement data to predict patient outcomes and intervention needs, such as in-hospital mortality[3] and circulatory failure[4] at ICU. Here we followed the feature selection and preprocessing strategies introduced by MIMIC-Extract[5] pipeline, a robust benchmark to process clinical structured data for machine learning research, providing 104 curated and aggregated clincal variables to construct patient time-series. Those features are:

01-04) Alanine aminotransferase; Albumin; Albumin ascites; Albumin pleural;

05-08) Albumin urine; Alkaline phosphate; Anion gap; Asparate aminotransferase;

09-12) Basophils; Bicarbonate; Bilirubin; Blood urea nitrogen;

13-16) Calcium; Calcium ionized; Calcium urine; Cardiac Index;

17-20) Cardiac Output fick; Cardiac Output Thermodilution; Central Venous Pressure; Chloride;

21-24) Chloride urine; Cholesterol; Cholesterol HDL; Cholesterol LDL;

25-28) CO2; CO2 (ETCO2, PCO2, etc.); Creatinine; Creatinine ascites;

29-32) Creatinine body fluid; Creatinine pleural; Creatinine urine; Diastolic blood pressure;

33-36) Eosinophils; Fibrinogen; Fraction inspired oxygen; Fraction inspired oxygen Set;

37-40) Glascow coma scale total; Glucose; Heart Rate; Height;

41-44) Hematocrit; Hemoglobin; Lactate; Lactate dehydrogenase;

45-48) Lactate dehydrogenase pleural; Lactic acid; Lymphocytes; Lymphocytes ascites;

49-52) Lymphocytes atypical; Lymphocytes atypical CSL; Lymphocytes body fluid; Lymphocytes percent;

53-56) Lymphocytes pleural; Magnesium; Mean blood pressure; Mean corpuscular hemoglobin;

57-60) Mean corpuscular hemoglobin concentration; Mean corpuscular volume; Monocytes; Monocytes CSL;

61-64) Neutrophils; Oxygen saturation; Partial pressure of carbon dioxide; Partial pressure of oxygen;

65-68) Partial thromboplastin time; Peak inspiratory pressure; pH; pH urine;

69-72) Phosphate; Phosphorous; Plateau Pressure; Platelets;

73-76) Positive end-expiratory pressure; Positive end-expiratory pressure Set; Post Void Residual; Potassium;

77-80) Potassium serum; Prothrombin time INR; Prothrombin time PT; Pulmonary Artery Pressure mean;

81-84) Pulmonary Artery Pressure systolic; Pulmonary Capillary Wedge Pressure; Red blood cell count; Red blood cell count ascites;

85-88) Red blood cell count CSF; Red blood cell count pleural; Red blood cell count urine; Respiratory rate;

89-92) Respiratory rate Set; Sodium; Systemic Vascular Resistance; Systolic blood pressure;

93-96) Temperature; Tidal Volume Observed; Tidal Volume Set; Tidal Volume Spontaneous;

97-100) Total Protein; Total Protein Urine; Troponin-I; Troponin-T;

101-104) Venous PvO2; Weight; White blood cell count; White blood cell count urine;

We refer readers to the original paper for more information on the data completeness and preprocessing procedures such as unit conversion and outlier handling. For each clinical variable at a specific time step (an hourly bin), the mean value of the recordings in that hourly bin and the mask to indicate whether the value is imputed or not were then used as features. In our experiment, we used data in the first 24 hours of ICU stay as input, thus resulting in 24 time steps, each with 208 (104×2) features. Missing values were first imputed in a carry-forward manner, where the most recent measured values were used to fill the missing ones. If there are no previous values in this case, the missing values would be set to the mean for the individual. Finally, if a variable was never observed, the value was set to the global mean in the training set during the cross validation (for example, global mean in the 4/5 of the data).

The preprocessed features were processed by a single layer LSTM and its last hidden state (equivalent to $\mathbf{O}_T$) was fed to a classifier to predict the DRG code. The setting to train this model was similar to the text-based model, where the cross entropy loss was minimized to find the most likely DRG target for each sample. The implementation details are introduced in the next section.

Supplementary Note 2. Implementation and hyper-parameter setting

We conducted all the experiments using PyTorch. Both text-based and measurement-based models were trained with Adam optimizer with an early stopping setting, where the training stopped if there was no improvement in micro-averaged F1 score performed on the validation set for 10 consecutive epochs. Since the training of neural net models is stochastic and we implemented early stopping, there is a need for a validation set to indicate when the model is sufficiently trained. To address this, we used 10% of the data during training as an early-stopping set to guide training. For example, when optimizing the hyper-parameters in each of the five folds of cross validation, 20% of the cross validation data was used for model selection and 80% was used to train the model, among which 10% (or 8% of the cross validation data, or 7.2% of the whole dataset that includes the hold-out test set) was actually used as an inner validation set to implement early stopping. Similarly, when the model was retrained using all cross validation data after the best hyper-parameter setup was determined, 10% of the data (or 9% of the whole dataset) was used for early stopping and was not seen by the model in learning.

For hyper-parameters searching, it was impossible to tune all possible settings given the wide range of hyper-parameters and the computational complexity. Therefore, for text-based model, we referenced to the settings of the original implementation of CAML model and tuned a limited combinations of learning rate, filter size, number of filters, dropout rate, and weight decay for each fold of cross validation for each DRG dataset. For LSTM, we tuned learning rate, weight decay, dropout rate, hidden size of the RNN, but also and batch size. The batch size for text-based model was fixed to 32. More implementation details can be found in the released repository.

Supplementary Method 3. Modeling with regressional objective

If we have no interest to learn about the DRG group and only care about the final cost or DRG weight, it is possible to bypass the classification step and to predict the weight directly as a regression problem. Here we described our experiments to model DRG relative weights using CAML, where the label in each sample was changed from the DRG code to DRG weight.

Following the denotations in previous sections, $\mathbf{O}_T$ is the hidden state extracted by CAML to represent the input clinical text. Instead of feeding it to a classifier, $\mathbf{O}_T$ was further processed by two more feed-forward layers with ReLU activation, with the first one having 100 hidden states and last layer having only one hidden state, which outputs a real number, $\hat{rw}^a = MLP(\mathbf{O}_T)$, as result. To train the model, we minimized Mean Square Error (MSE) as the objective, where

$$MSE(D, y, \theta) = \frac{1}{A} \sum_{i=1}^{|Y|} ||rw^a - \hat{rw}^a||_2 \tag{4}$$

and A is the total number of patient stays in a training batch.

For evaluation, we calculated MAE (Mean Average Error), RMSE (Root Mean Square Error), and R2 to show how accurate a model is in assessing DRG weight. As a comparator, we listed the model for DRG classification as a comparator where weights were mapped to the predicted DRGs. Based on results in Supplementary Table 1, we could observe that it is feasible to directly predict cost weight in a regression manner, bypassing DRG prediction, though the relatively high errors again showed the challenge to estimate cost for each individual patient accurately. The performances of directly modeling payment weight were in fact superior than translating weight from predicted DRG codes, indicating the capacity of the model to further fit to specific cost information in the data. However, this strategy was unable to provide DRG groupings and thus made the prediction less interpretable and potentially less desirable in certain scenarios. In addition, obtaining weights by mapping from classification allows perfectly predicted cases (when the model makes right classification) and could contribute to offsetting over- and under-predicted weights. Nevertheless, Supplementary Table 1 demonstrated the potential to extend the experimental setup of modeling routine clinical data to predict DRG-based cost to payment system other than DRG when past payment records can be obtained.

Supplementary Table 1. Early weight prediction using text-based model

	Model	MACRO-AUC	MAE	RMSE	R2
MS-DRG	CAML classification	0.871 (0.011)	1·538(0·023)	3·158(0·044)	-0·282(0·036)
	CAML regression	—	1.282(0.010)	2.378(0.030)	0.273(0.018)
APR-DRG	CAML classification	0.884 (0.003)	1·726(0·023)	3·449(0·070)	-0·154(0·046)
	CAML regression	—	1.499(0.008)	2.859(0.013)	0.208(0.007)

Results on MS-DRG and APR-DRG. CAML classification refers to obtaining weight result by mapping the predicted DRGs from the DRG prediction model. Macro-AUC is presented for CAML classification, mapping the results presented in the main manuscript. All results are presented in mean (standard deviation) averaged across five folds. DRG: Diagnostic Related Group; MS: Medicare Severity; APR: All Patient Refined; CAML: Convolutional Attention for Multi-Label classification.

Supplementary Table 2. Absolute CMI errors across HPA

HPA	Absolute CMI Error	
	MS-DRG	APR-DRG
-24	65·34% (3·01%)	72·03% (0·53%)
-18	62·80% (2·99%)	70·31% (0·55%)
-12	60·14% (2·88%)	68·02% (0·65%)
-6	57·50% (2·84%)	64·06% (0·71%)
0	39·04% (3·04%)	40·79% (2·19%)
6	16·32% (2·52%)	18·29% (3·12%)
12	7·53% (1·87%)	14·82% (2·68%)
18	4·95% (1·50%)	13·97% (2·18%)
24	2·41% (1·08%)	12·79% (2·31%)
30	1·26% (1·22%)	12·56% (2·31%)
36	1·23% (0·88%)	12·51% (2·33%)
42	1·02% (0·74%)	11·95% (2·48%)
48	1·52% (0·62%)	11·70% (2·55%)

Here are the detailed CMI errors used to plot Figure 1 in the main manuscript, for the two cohorts respectively. CMI: Case Mix Index; HPA: Hour Post Admission.

Supplementary Table 3. Early DRG prediction using structured clinical measurements

		DRG set	MACRO-AUC	MICRO-AUC	MACRO-F1	MICRO-F1	Number of DRG targets	Number (%) of hospital stays
MS-DRG	LSTM with clinical measurements	All DRGs	0.819 (0.009)	0.940 (0.004)	0.041 (0.008)	0.183 (0.011)	369	1977.0 (100.0)
		MDC 05	0.834 (0.005)	0.972 (0.003)	0.066 (0.017)	0.258 (0.015)	67	522.0 (26.4)
		MDC 01	0.692 (0.026)	0.946 (0.002)	0.044 (0.009)	0.206 (0.015)	52	320.0 (16.2)
		MDC 04	0.767 (0.024)	0.961 (0.009)	0.059 (0.008)	0.179 (0.034)	34	219.0 (11.1)
		Top 80% cases	0.879 (0.006)	0.973 (0.005)	0.108 (0.012)	0.228 (0.012)	131	1581.0 (80.0)
		Top 50 DRGs	0.905 (0.003)	0.985 (0.004)	0.221 (0.008)	0.319 (0.018)	50	1083.0 (54.8)
		Top 30 DRGs	0.916 (0.004)	0.988 (0.003)	0.298 (0.007)	0.383 (0.023)	30	842.0 (42.6)
APR-DRG	LSTM with clinical measurements	All DRGs	0.838 (0.017)	0.946 (0.006)	0.037 (0.004)	0.160 (0.012)	517	2747.0 (100.0)
		MDC 05	0.843 (0.023)	0.975 (0.006)	0.073 (0.007)	0.223 (0.011)	87	733.0 (26.7)
		MDC 01	0.775 (0.006)	0.958 (0.008)	0.058 (0.003)	0.171 (0.022)	63	424.0 (15.4)

		MDC 04	0.742 (0.030)	0.947 (0.010)	0.063 (0.004)	0.145 (0.016)	47	319.0 (11.6)
		Top 80% cases	0.897 (0.012)	0.974 (0.006)	0.109 (0.007)	0.204 (0.013)	180	2197.0 (80.0)
		Top 50 DRGs	0.919 (0.007)	0.986 (0.004)	0.236 (0.013)	0.310 (0.020)	50	1285.0 (46.8)
		Top 30 DRGs	0.913 (0.007)	0.989 (0.003)	0.245 (0.008)	0.343 (0.022)	30	976.0 (35.5)

Detailed results on applying LSTM to model structured clinical measurements to predict early DRG on the first day of ICU admission. This table expands the results of LSTM using structured data presented in the main manuscript.

Supplementary Method 4. Text-based DRG Prediction with BERT and ClinicalBERT

Here we provide results of applying pre-trained models on the early DRG prediction based on clinical notes. We examined BERT-base model (12 layer, uncased)[6], a Transformer-based model that is pre-trained on large quantities of text, and fine-tuned the model to predict DRGs based on progress notes. We followed our previous preprocessing steps to remove de-identification placeholders from the concatenated reports and applied BERT tokenizers to tokenize the text string, ensuring the original text inputs are the same across different modeling. Given the BERT capacity, the number of input tokens is limited to 512, including two special tokens [CLS] and [SEP] that are at the start and the end of an input sequence. The [CLS] token was fed to a feed-forward layer to produce the model output in both training and testing, following BERT's normal fine-tuning pattern for sequence classification. We again trained (or fine-tuned) five different models in each of the five-fold cross-validation experiments, with the difference that we did not change the hyper-parameters but instead follow the recommended practice for classification-oriented fine-tuning[6,7], which is to set learning rate to 2e-5 and batch size to 16. In this case, each of the five BERT models saw slightly different training data (as 10% was randomly sampled for early stopping), but it is the same training data with its CAML counterpart. In short, we ensured that CAML and BERT were compared fairly using the same training and testing dataset splits, though BERT consumes shorter input compared to CAML. Finally, we also fine-tuned the domain adapted version of BERT, ClinicalBERT (cased)[8], which was further pre-trained on biomedical text (initialized from BioBERT) and clinical notes. We refer readers to the original publications for more details on BERT and ClinicalBERT.

Supplementary Table 4. Early DRG prediction with BERT-based NLP models

		MACRO-AUC	MICRO-AUC	MACRO-F1	MICRO-F1
MS-DRG	CAML	0.871 (0.011)	**0.956 (0.002)**	**0.084 (0.008)**	**0.270 (0.006)**
	BERT	0.875 (0.006)	0.953 (0.002)	0.067 (0.005)	0.255 (0.004)
	ClinicalBERT	**0.882 (0.007)**	**0.956 (0.002)**	0.079 (0.012)	0.266 (0.003)
APR-DRG	CAML	0.884 (0.003)	0.963 (0.001)	**0.069 (0.008)**	**0.244 (0.005)**
	BERT	0.902 (0.002)	0.965 (0.000)	0.048 (0.006)	0.229 (0.004)
	ClinicalBERT	**0.908 (0.003)**	**0.967 (0.001)**	0.054 (0.005)	0.233 (0.004)

The table presents the results of the CNN-based model (CAML) and the pre-trained Transformer-based models (BERT and ClinicalBERT) on predicting all test DRGs in both MS-DRG and APR-DRG cohorts, which contain 1977 cases (369 unique labels) and 2747 cases (517 labels), respectively. The results were summarized in mean (standard deviation) over the performances of five models on the hold-out test set. Best performances are highlighted in boldface.

Supplementary References

1 Mullenbach J, Wiegreffe S, Duke J, Sun J, Eisenstein J. Explainable prediction of medical codes from clinical text. In: *Proc NAACL* 2018; **1**: 1101–11.

2 Zhang Y, Chen Q, Yang Z, Lin H, Lu Z. BioWordVec, improving biomedical word embeddings with subword information and MeSH. *Sci Data* 2019; **6**: 52.

3 Rajkomar A, Oren E, Chen K, *et al.* Scalable and accurate deep learning with electronic health records. *npj Digit Med* 2018; **1**. DOI:10.1038/s41746-018-0029-1.

4 Hyland SL, Faltys M, Hüser M, *et al.* Early prediction of circulatory failure in the intensive care unit using machine learning. *Nat Med* 2020; **26**: 364–73.

5 Wang S, McDermott MBA, Chauhan G, Ghassemi M, Hughes MC, Naumann T. MIMIC-Extract: A Data Extraction, Preprocessing, and Representation Pipeline for MIMIC-III. In: *Proc ACM CHIL* 2020: 222–235.

6 Devlin J, Chang MW, Lee K, Toutanova K. BERT: Pre-training of Deep Bidirectional Transformers for Language Understanding. In: *Proc NAACL* 2019; **1**: 4171-4186.

7 Adhikari A, Ram A, Tang R, Lin J. DocBERT: BERT for Document Classification. arXiv preprint arXiv:1904.08398. 2019 Apr 17.

8 Alsentzer E, Murphy JR, Boag W, Weng WH, Jin D, Naumann T, McDermott M. Publicly available clinical BERT embeddings. In: *Proc Clinical NLP Workshop* 2019: 72-78.
