# Supplementary material for: Early prediction of diagnostic-related groups and estimation of hospital cost by processing clinical notes
Source: NPJ Digit Med. 2021 Jul 1;4:103. doi: 10.1038/s41746-021-00474-9 (PMC8249417; doi:10.1038/s41746-021-00474-9)
Supplement: Supplementary file 2 — Reporting Summary [file 41746_2021_474_MOESM2_ESM.pdf]

nature research

Corresponding author(s): Karin Verspoor

Last updated by author(s): Mar 9, 2021

Reporting Summary

Nature Research wishes to improve the reproducibility of the work that we publish. This form provides structure for consistency and transparency in reporting. For further information on Nature Research policies, see our Editorial Policies and the Editorial Policy Checklist.

Statistics

For all statistical analyses, confirm that the following items are present in the figure legend, table legend, main text, or Methods section.

n/a	Confirmed	
☐	☒	The exact sample size (*n*) for each experimental group/condition, given as a discrete number and unit of measurement
☐	☒	A statement on whether measurements were taken from distinct samples or whether the same sample was measured repeatedly
☐	☒	The statistical test(s) used AND whether they are one- or two-sided<br>*Only common tests should be described solely by name; describe more complex techniques in the Methods section.*
☒	☐	A description of all covariates tested
☒	☐	A description of any assumptions or corrections, such as tests of normality and adjustment for multiple comparisons
☐	☒	A full description of the statistical parameters including central tendency (e.g. means) or other basic estimates (e.g. regression coefficient) AND variation (e.g. standard deviation) or associated estimates of uncertainty (e.g. confidence intervals)
☒	☐	For null hypothesis testing, the test statistic (e.g. *F*, *t*, *r*) with confidence intervals, effect sizes, degrees of freedom and *P* value noted<br>*Give P values as exact values whenever suitable.*
☒	☐	For Bayesian analysis, information on the choice of priors and Markov chain Monte Carlo settings
☒	☐	For hierarchical and complex designs, identification of the appropriate level for tests and full reporting of outcomes
☒	☐	Estimates of effect sizes (e.g. Cohen's *d*, Pearson's *r*), indicating how they were calculated

Our web collection on statistics for biologists contains articles on many of the points above.

Software and code

Policy information about availability of computer code

Data collection	We analyzed a public dataset from a major medical center in the United States on acute patients. The MIMIC-III dataset can be requested https://physionet.org/content/mimiciii/1.4/, which requires signed safe usage agreement and for research-only.
Data analysis	All analyses, including preprocessing, training and evaluation in this work were performed using Python 3.6, with packages including pytorch and scikit-learn. The scripts are made available with accompanied documentation at https://github.com/JHLiu7/EarlyDRGPrediction.

For manuscripts utilizing custom algorithms or software that are central to the research but not yet described in published literature, software must be made available to editors and reviewers. We strongly encourage code deposition in a community repository (e.g. GitHub). See the Nature Research guidelines for submitting code & software for further information.

Data

Policy information about availability of data

All manuscripts must include a data availability statement. This statement should provide the following information, where applicable:

- Accession codes, unique identifiers, or web links for publicly available datasets
- A list of figures that have associated raw data
- A description of any restrictions on data availability

Access to MIMIC-III can be requested at https://physionet.org/content/mimiciii/1.4/, which requires signed safe usage agreement and for research-only.

Field-specific reporting

Please select the one below that is the best fit for your research. If you are not sure, read the appropriate sections before making your selection.

☒ Life sciences ☐ Behavioural & social sciences ☐ Ecological, evolutionary & environmental sciences

For a reference copy of the document with all sections, see nature.com/documents/nr-reporting-summary-flat.pdf

Life sciences study design

All studies must disclose on these points even when the disclosure is negative.

Sample size	Sample size was determined by including patients over 18 years of age, patients having only one ICU visit per hospital stay, and stays at least one clinical note charted before the 48th hour after ICU admission. Each hospital stay should be assigned to one diagnostic-related group (DRG) of one of the two DRG systems examined in the study, namely MS-DRG and APR-DRG. These criteria were imposed on MIMIC-III, which collects inpatient data associated with over 40,000 patients from 2001 to 2012. These resulted in 16,484 patients for MS-DRG and 22,518 patients for APR-DRG.
Data exclusions	There were no additional exclusions beyond the inclusion criteria described above.
Replication	The performance of the machine learning model was evaluated on an hold-out, unseen test set, which accounts for 10% of the total data for each of the MS-DRG and APR-DRG cohorts. Source code to reproduce the results demonstrated in the paper is released on open repository.
Randomization	The 90% of data used to derive the model were further split into five subsets to perform five-fold cross-validation. After selecting best performing hyper-parameters for the machine learning model, each model was retrained under each fold of the cross-validation setting, resulting in five different models that had been initialized differently and had seen different training data. Each of such models were evaluated finally on the hold-out test set, and we report the average performance across the five models on this same test set.
Blinding	All patients were included into the cohort and were split into train and test sets randomly. The investigators were blinded to the splitting.

Reporting for specific materials, systems and methods

We require information from authors about some types of materials, experimental systems and methods used in many studies. Here, indicate whether each material, system or method listed is relevant to your study. If you are not sure if a list item applies to your research, read the appropriate section before selecting a response.

Materials & experimental systems

n/a	Involved in the study	
☒	☐	Antibodies
☒	☐	Eukaryotic cell lines
☒	☐	Palaeontology and archaeology
☒	☐	Animals and other organisms
☒	☐	Human research participants
☒	☐	Clinical data
☒	☐	Dual use research of concern

Methods

n/a	Involved in the study	
☒	☐	ChIP-seq
☒	☐	Flow cytometry
☒	☐	MRI-based neuroimaging
